# Proceedings of the Pathological Society of Philadelphia

**Published:** 1858-11

**Authors:** 


ART. III.—*Proceedings of the Pathological Society of Philadelphia.*

Reported by the SECRETARY.

WEDNESDAY EVENING, September 8th, 1858.

The President, DR. GROSS, in the chair.

*Abnormal Size of the Groove for the Meningeal Artery, and Dilatation at its termination.*—DR. AGNEW presented a skull with a very large groove for the great meningeal artery of the right side, and near the vertex at the termination of the groove, a depression of sufficient depth to receive the end of the middle finger. He remarked that the Society would remember that on a former occasion he had exhibited a skull-cap having a similar groove and depression, but as no history could be obtained all explanations offered were only problematical. It was believed, however, to be due to an aneurismal dilatation of the vessel. The present specimen he thought might furnish a satisfactory explanation of the condition in question. The artery though unusually large, does not exhibit any positive evidence of dilatation, and near the vertex the main trunk breaks up into a great number of small parallel branches, with unusual groups of Pacchionian bodies placed between. Wherever these bodies are found there are usually little pits in the inner table of the cranium for their reception. In this case the extraordinary size of the groups placed among the numerous meningeal branches, derived from the main arterial trunk, will account for the large depression.

*Tubercular Abscess of Right Kidney, and Chronic Inflammation of the Bladder, with destruction of its Mucous Membrane.*—DR. GROSS exhibited a diseased bladder and kidney removed from John Anthony, aged fifty years, a resident of Carbon County, Pennsylvania, who had been suffering for four years with symptoms of vesical and renal disease. He consulted Dr. Gross early in August last; he was much emaciated, and became easily fatigued from the slightest exercise. He complained of pain in the right loin, and very severe pain in the region of the prostate gland. When first seen he stated that he had come to be treated for stricture of the urethra; but upon introducing a sound, the canal was found to be completely pervious, although somewhat tender; the sensation imparted by the examination was such as to induce the belief that the muscular coat of the bladder was hypertrophied and columniform. Mr. Anthony passed his water during the day about once every hour, and was obliged to get up seven or eight times during the night. His urine, which was offensive and scanty, was passed in a small stream, with much pain, and contained pus. The pain in the region of the prostate was so severe as to require the use of opiates. His appetite was bad, and he complained of cough, with expectoration. The treatment consisted in the administration of the copaiba mixture with morphia to relieve pain, and he was ordered brandy and a nourishing diet; but he had no appetite for anything, and died in about two weeks from utter exhaustion.

Upon examination, seven hours after death, the right lung was found to be adherent to the pleura throughout the whole posterior aspect of the superior lobe, and in the apex and back portion of the same lobe a large tubercular cavity was found, as well as several smaller ones. The mitral tricuspid and aortic valves were thickened, and in some portions opaque; in other respects the heart was normal. Some of the mesenteric glands were enlarged. The left kidney was healthy, but the right was enlarged, and firmly adherent to its investing membrane. The substance of the gland was in some places so thin as to show the presence of pus beneath. Upon a section being made, this fluid gushed out to the amount of about two ounces; it was offensive, and bore all the distinctive features of being tubercular. The cortical portion of the kidney was much discolored. The ureter was but little enlarged, and contained some matter. Its mucous coat was slightly inflamed, and presented a capilliform injection.

Upon removing the bladder the index finger perforated the very thin walls at its apex, allowing the escape of a large quantity of pus into the pelvic cavity. The mucous membrane had completely disappeared, leaving the muscular coat entirely denuded, and presenting a deep maroon appearance. A cavity containing pus was found in the left lobe of the prostate gland, and the prostatic portion of the urethra was without a mucous covering. The bladder contained about six ounces of purulent matter mixed with a small quantity of urine.

DR. GROSS believed that such complete destruction of the mucous coat of the bladder was always the result of tubercular disease. He had examined several specimens which had confirmed him in this opinion. The deposits of tubercle taking place in the submucous tissue give rise, by their softening, to the extensive destruction witnessed in the organ.

DR. STILLÉ inquired whether there had ever been gonorrhœa. The morbid appearances, both in the bladder and kidney, did not seem to him to be of necessity tubercular; they might have been caused by a cystitis or nephritis, and the tubercular disease of the lung be independent of the affection of the urinary organs. No distinct tubercles in the kidney were perceived.

DR. GROSS replied that a disease of the urethra had not at any time existed.

DR. KELLER spoke of a case of tuberculosis of both kidneys which had come under his care for a supposed vesical affection. The bladder was, however, found to be perfectly healthy, but the normal structure of both kidneys was entirely effaced. In one, the disease with what remained of the renal tissue formed a confused mass of broken down texture, in the other, the isolated gray tubercles were distinct.

*Cystic Growths on Intestines.*—DR. PACKARD stated that the subject in whom these growths were found was a man, aged thirty-seven, a patient in the Pennsylvania Hospital for nearly a year, on account of ascites, for which he was eight or nine times tapped; cirrhosis of the liver was suspected.

The abdominal cavity, when laid open at the autopsy, showed abundant traces of chronic peritonitis, the serous membrane having deposits of lymph scattered over its entire surface. The liver particularly was encased in a thick layer of false membrane, but its size was not notably diminished, nor was there anything else abnormal about it, except that its tissue was less easily torn than is usually the case.

The intestines seemed entirely healthy, but at different points along the canal were attached cystic growths, over a dozen in number; these growths were of various sizes, from that of a marble to that of an orange, the largest being situated in the great omentum, close to the transverse colon. They had no connection, vascular or otherwise, with the bowel, merely lying in the sub-serous tissue. One of the smaller ones only was subjected to a careful examination: it contained a clear-yellow liquid, somewhat ropy, like synovia, and stretching in a wavy manner through this were several flocculent masses or bands, like imperfectly-formed septa. The liquid, placed under the microscope, was entirely homogeneous, but here and there one or two nucleated granular cells were seen floating in it. The flocculent bands alluded to were found to have a dimly-fibrillated or nebulous appearance.

DR. HEWSON reported the death of the patient whose thigh-bone he had exhibited to the Society. (See Proceedings of Dec. 23d, 1857.) The wound had nearly healed, but a troublesome diarrhœa set in about three months after the operation, which exhausted the patient. He died on the 16th of July.

---

WEDNESDAY EVENING, September 22d, 1858.

The President, DR. GROSS, in the Chair.

*Abscess of the Brain following Fracture of the Skull.*—DR. HALL exhibited for DR. HUTCHINSON a fractured skull and an abscess of the brain. The patient, R. W., aged twenty-three, colored, was admitted into the Pennsylvania Hospital on Friday, September 10th, with contused wounds of the scalp and two wounds of the face, which appeared to have been made by penetrating instruments. Through one of the wounds of the scalp a depressed fracture of the parietal bone was discoverable; the zygoma also was found to be fractured. The patient was unable to give any particulars of himself, and no satisfactory account of the accident has been obtained. He was able to walk, but was, during and after the examination, so exceedingly restless, that he had to be confined. His pupils were sluggish, but neither dilated nor contracted; the pulse was ninety-four. He was trephined the next day, and the depressed portion of bone raised, but he never fully recovered his power of speech, although his intelligence increased, so much so that he put his tongue out when told to do so, and appeared to take an interest in things around him. His fæces, however, passed involuntarily from him, and his urine had to be drawn off by a catheter. Four days after the operation he had a chill, which was very slight, and not repeated. But his symptoms became again worse, and he gradually relapsed into a comatose state. Just before his death, on the morning of the 22d, abscesses formed about his ankles, which discharged pus and a quantity of fetid gas. Upon making a post-mortem examination, the brain was perceived to be much softened, and a large abscess existed immediately beneath the point at which he had been trephined. The fracture was found to extend down to the base of the skull, involving the sphenoid and temporal bones.

*Metastatic Abscesses of the Liver following an Injury of the Head.*—DR. HALL next exhibited for DR. HUTCHINSON, a liver with several abscesses, from the body of Edward Morris, aged fifty-seven years, seaman, who was admitted into the Pennsylvania Hospital on Thursday, the 2d of September, with a contused wound of the scalp over the left parietal bone, and one on the same side of face, beneath the orbit. Neither appeared to be serious, and there was nothing in his condition to occasion any alarm until Sunday, September 19th.

On Friday he complained of pain in his head, but this was not sufficient to confine him to bed. On the same day he had a chill, which, as he formerly suffered from an intermittent, was attributed to that cause.

On Saturday he complained of pain in his abdomen, which was relieved by a mustard plaster. He had an old rupture, but there was no reason to suspect any strangulation.

Finding on Sunday that the patient had retained his water, about f℥iv of a yellowish liquid was drawn off, which did not possess, in the slightest degree, the smell of urine.

On Monday a little blood was discharged through the catheter. During Sunday night and Monday, he had spasms of the muscles of the right side of the body, and in a less degree of the left side. He died on Tuesday morning at two o'clock; the autopsy was made on the same day at 2 P.M.

There was no fracture of the skull, but immediately beneath the spot upon which he had been struck was a deposit of pus, and within the membranes a deposit of lymph. The brain was very much congested. In the liver several abscesses were detected, and in the kidneys some pus. The bladder was very much contracted, and contained clots of blood.

This specimen led to an animated discussion, mainly carried on by Drs. Keating, Hewson, and Woodward, in which the different prevalent theories concerning the formation of metastatic abscesses were reviewed.

*Tubercles in the Peritoneum and in the Liver.*—The Secretary presented, for DR. H. LENOX HODGE, a specimen of tubercular peritonitis, with the subjoined history:—

Patrick Bannon, aged fifty-four years, a native of Ireland, was admitted into the Pennsylvania Hospital on the 6th of August, for dropsy. He could not give a very accurate account of his disease, but stated, in reply to our questions, that until he had taken this affection he was well as anybody, that he had not been able to work since spring, that now he could eat nothing, and was obliged to drink all the time. Upon examination, the abdomen was found tense, but not very much distended; fluctuation, although not very evident, could be detected; there were vague pains throughout the part, especially on motion; the urine contained no albumen, and showed no evidence of disease; the heart appeared healthy upon percussion and auscultation; the liver certainly was not enlarged, and perhaps did not cause dullness over quite as large a space as usual. After noticing these symptoms, Dr. Gerhard said that the case was probably one of chronic peritonitis. Under the influence of diuretics, such as cream of tartar, juniper berries, squill, and digitalis, the dropsical symptoms lessened and soon disappeared. After a time, upon the application of counter-irritants, the pains left the abdomen. Thus the patient became more comfortable; but he had no appetite, and he grew more and more emaciated. A slight cough that he had had before entering the hospital increased; evidences of tubercles and cavities were detected in the lungs; and gradually becoming weaker and weaker, he died yesterday, (the 21st,) in the morning.

Autopsy.—The lungs contained tubercles at the upper portion, especially of the right lung, where there was a cavity, and also one at its very base. There were very few pleuritic adhesions, and these chiefly at the lower portions. The heart was healthy, the pulmonary arteries considerably dilated. Upon opening the abdominal cavity, the peritoneum was found much thickened, and the abdominal walls everywhere studded with tubercles; the mesenteric glands were much enlarged. Liver about the normal size, but containing, in the left lobe especially, tubercles. Kidneys were healthy. The spleen contained tubercles in its capsule, though not in its substance.

*Ulcer of the Stomach.*—DR. KEATING presented a specimen of perforating ulcer of the stomach, and gave the following history:—

Mrs. L., aged fifty-one, of sallow complexion and spare habit, had consulted me at various intervals during the last four years, in reference to a pain which she now and then experienced over the cardiac orifice of the stomach. She occasionally suffered from violent pyrosis, cardialgia, and other symptoms of dyspepsia, and became at times exceedingly emaciated. Having in connection with this pain constant palpitations of the heart, she was convinced that her affection originated from some cardiac disorder. During the month of February last, she was occasionally troubled with rejection of her food about an hour after eating; but this, as well as her other dyspeptic symptoms, yielded rapidly to treatment.

In March last, I was sent for in haste to visit her on account of a profuse vomiting of a chocolate-colored fluid, in all about a pint and a half. I submitted a portion of this fluid to my friend Dr. Da Costa for an examination under the microscope; he reported the existence of broken-down blood corpuscles in abundance, sarcinæ ventriculi, and mucous membrane in a state of degeneration. This hemorrhage had been preceded by a great increase of pain over the left side, corresponding with the cardiac orifice of the stomach. Upon careful examination, I could not discover the existence of a tumor in any portion of the stomach or abdominal cavity. The hemorrhage soon yielded to treatment, but it was followed by a constant rejection of the food taken during the day. Sometimes the vomiting occurred two hours after each meal; at other times twelve hours would elapse without any rejection of food, and then suddenly about daybreak profuse emesis would take place, bringing up in a partially digested form the engesta of several meals. This state of things continued with but little variation for four weeks, although at times the symptoms would lull, and apparently yield to treatment, so as to induce hope that the disease might be cured. At one period she was nearly three weeks without vomiting, and began to gain flesh. Another hemorrhage set in, and was followed by the previous train of morbid symptoms.

Her appearance at times indicated so strongly a malignant disease, that Dr. Pepper, who visited her in consultation, feared with me that the ulceration might be malignant, especially as she had had a sister who died of scirrhus of the rectum. These fears, however, were dispelled by our examination for a tumor, no traces of which could be detected. The pain continued over the left portion of the stomach, the vomiting increased, until, on the 16th of the last month, symptoms of perforation set in, and death followed in twelve hours.

Post-mortem examination revealed a healthy state of all the organs save the stomach; in which was found, about an inch from the cardiac orifice, on the greater curvature, a perforated spot, with thick indurated edges; about half an inch from this ulcer there was a partial softening of the stomach, and evident signs of another ulcer forming. The remaining portions of the stomach were in a healthy condition. An examination of the ulcerated part by Dr. Da Costa, under the microscope, gave no evidence of malignant disease; and hence, although situated near the cardiac orifice and along the greater curvature, the disease was undoubtedly a chronic perforating ulcer, with a constant deposit on its edges during the gradual process of perforation.

The death in these cases usually ensues from hemorrhage, emaciation, or perforation. In her case the hemorrhage was always immediately controlled by the tincture of the perchloride of iron; the emaciation was not excessive, for at the post-mortem examination large deposits of fat were found under the abdominal muscles, and in different portions of the abdomen. In reflecting upon the therapeutics of such cases, I believe that the greatest chances of success are to be found in a continued opiate treatment, with an allowance of just sufficient nutriment to support life; the stomach might thus be placed in the condition of throwing out bands of lymph, and of protecting itself from perforation and effusion of its contents. Undoubtedly such a method of treatment affords more hope in the chronic perforating ulcer, than in the acute with its sharp edges.

The question which presents itself for solution is, whether these ulcers are simple local lesions, or whether they may be a local expression of a general cachectic condition of the whole system? In this case, a point of interest lies in the fact that the patient was of an age at which ulcer of the stomach is certainly not often met with; nor is, indeed, the disease in this country at all frequent. Judging from the few cases which have been reported, its occurrence must be far rarer than in England, or on the Continent of Europe.

